# Outcomes of newborn hearing screening at an academic secondary level hospital in Johannesburg, South Africa

**DOI:** 10.4102/sajcd.v68i1.741

**Published:** 2021-01-27

**Authors:** Jacqueline K. Bezuidenhout, Katijah Khoza-Shangase, Tim De Maayer, Renate Strehlau

**Affiliations:** 1 Department of Paediatrics, Faculty of Health Sciences, University of the Witwatersrand, Johannesburg, South Africa; 2 Department of Speech Pathology and Audiology, Faculty of Humanities, University of the Witwatersrand, Johannesburg, South Africa

**Keywords:** otoacoustic emission, newborn hearing screening, refer rates, risk factors, public healthcare, outcomes

## Abstract

**Background:**

The Health Professions Council of South Africa (HPCSA) issued early hearing detection and intervention guidelines, which has universal newborn hearing screening (UNHS) as one of the important goals. Despite established evidence of the importance of UNHS globally, there has been no mandated formalised and standardised implementation as yet in South Africa.

**Objectives:**

The aim of this study was to describe the outcomes of newborn hearing screening (NHS) in an academic secondary level hospital in Johannesburg, South Africa.

**Methods:**

This was a prospective non-experimental feasibility study over a 3-month period, involving conducting hearing screening of 121 neonates. Audiologists conducted a risk factor assessment, otoscopic examinations and distortion product otoacoustic emissions (DPOAEs) screening on each neonate, with follow-up appointments for re-screening and diagnostic audiological assessments for all neonates with *refer* findings. Data were analysed using STATA intercooled version 11^©^, through both descriptive and inferential statistics (Fisher’s exact test), with significance established where *p*-values less than 0.05 were considered statistically significant.

**Results:**

Of the 121 neonates screened, the majority (75%) were screened in the first 24 h of life. A high *refer* rate (47%) of the total sample was found on DPOAE screening. No maternal or neonatal risk factors were found to be significantly associated with *refer* findings.

**Conclusion:**

Findings contribute towards the existing evidence base that raises implications for successful implementation of NHS programmes in public healthcare in South Africa. Screening protocols need to consider the timing of screening, the measures and procedures adopted in the screening protocols, as well as the follow-up strategies.

## Introduction

The low- and middle-income world, where South Africa is located, is reported to be home to two-thirds of the world’s children with hearing impairment (Olusanya, Luxon, & Wirz, 2004). The prevalence of hearing impairment in South Africa is three to six in every 1000 live births, with the public healthcare sector recording the highest numbers of individuals affected (Swanepoel & Storbeck, 2008). This is one of the reasons why the Health Professions Council of South Africa (HPCSA, 2018), in carrying out its mandate to protect the public and guide the professions, recently published guidelines on early hearing detection and intervention (EHDI).

Currently, there remains a paucity of sufficient evidence regarding the current status of neonatal hearing screening programmes in South Africa both in the public and private healthcare sector. The available evidence indicates limited success with implementation of these programmes within the South African context (Maluleke, Khoza-Shangase, & Kanji, 2018; Swanepoel, Storbeck, & Friedland, 2009). Theunissen and Swanepoel (2008) reported that only 27% of public sector hospitals in South Africa were implementing any form of newborn hearing screening (NHS). These findings were not that far different from the private healthcare sectors, where resource constraints are not as prevalent as in the public healthcare sector (Khoza-Shangase, 2021b). In a national survey of the audiological services for diagnosis and intervention in the private healthcare sector in South Africa, findings revealed that there is significant delay in the overall diagnosis and provision of intervention for hearing impairment (Kanji & Khoza-Shangase, 2018b; Khoza-Shangase, Barratt, & Jonosky, 2010; Meyer, Swanepoel, & Le Roux, 2014).

Universal newborn hearing screening (UNHS) is when every newborn baby is screened for hearing impairment at birth. Currently, in South Africa, no legislation exists to implement UNHS and because of resource constraints, the approach to NHS has been targeted screening (HPCSA, 2018). Targeted hearing screening, where babies with risk factors for hearing loss are screened, has the risk of missing a significant number of infants with hearing impairment, and assumes the universality of the listed risk factors. However, Kanji (2018) strongly argues for the implementation of this as an interim measure within the South African context – if contextual responsiveness is adopted. Nonetheless, studies have shown that by screening only those infants considered ‘high-risk’, approximately 50% of infants with hearing impairment would be missed (Chu et al., 2003; Kanne, Schaefer, & Perkins, 1999). Khoza-Shangase (2021b) recommends this adoption of targeted NHS as a starting point or interim approach, particularly in a hospital setting, with inclusion of NHS at the first follow-up visit at midwife obstetric units for all babies, including those without risk factors as well as those who were born at home. This author believes that this approach respects both the documented evidence of established risk factors for hearing impairment and the contextual challenge of resource constraints.

Evidence suggests that in South Africa, where different types and levels of healthcare exist, NHS programmes have neither been standardised, nor have they been uniformly or universally implemented nationally (Khoza-Shangase & Kanji, 2021). In a study examining infant hearing screening in two South African provinces, findings indicated lack of formal, standardised and systematic EHDI implementation at all three levels of public healthcare (Khoza-Shangase, Kanji, Petrocchi-Bartal, & Farr, 2017). The authors of this study proposed that some of the reasons for the lack of EHDI included lack of equipment, budgetary constraints, human resource challenges, as well as lack of political mandate by the South African government. These findings have highlighted the need for ensuring that context-specific studies in infant and neonatal hearing screening are conducted to ensure that contextually relevant strategies are put in place, which allows for evidence-based practice (Khoza-Shangase et al., 2017).

Another recent study from the South African context which explored factors associated with follow-up return rate in a risk-based NHS programme found that addressing the challenges to implementation of NHS is imperative towards successful EHDI (Kanji & Khoza-Shangase, 2018b). In this study, 66.5% of the participants returned for repeat screening, and this follow-up return rate decreased to below 50% for follow-up diagnostic assessment. Various reasons for poor return rate were identified, with one of the key challenges contributing to nonattendance being changes in residential location. The authors suggest that strategic bookings of appointments for screening where there is improved alignment of hearing screening appointments with other medical follow-up services are key to successful implementation of neonatal screening (Kanji & Khoza-Shangase, 2018b).

Other factors influencing successful implementation of hearing screening within the South African context, particularly UNHS, include the insufficient number of audiologists available to provide screening, the high rate of false positive test results and the high rates of loss to follow-up (Bezuidenhout, Khoza-Shangase, De Maayer, & Strehlau, 2018; Khoza-Shangase, 2021b). Furthermore, the quadruple burden of disease that guides priorities within the South African healthcare sector places hearing impairment low as a risk priority. Conditions high on the priority list tend to be those deemed life-threatening such as human immunodeficiency virus (HIV), acquired immune deficiency syndrome (AIDS) and tuberculosis which are in the top five contributors of death in South Africa (Khoza-Shangase, 2021a; StatsSA, 2016).

Regardless of hearing impairment being placed low on the government’s priority list, sufficient evidence exists to support the importance of identifying it early and providing intervention by 6 months of age (HPCSA, 2018; Khoza-Shangase & Kanji, 2021). The year 2019 position statement from the Joint Committee on Infant Hearing (JCIH) advocates the EHDI 1:3:6 goals. These goals state that infants must have initial hearing screening done by 1 month of age, have their hearing status confirmed by 3 months of age and be receiving appropriate intervention by 6 months of age. Sufficient evidence exists proving the positive benefits of EHDI towards cognitive, linguistic, literacy and educational, social, and emotional development, with consequent positive vocational and thus financial outcomes (Dillon, Cowan, & Ching, 2013; Maluleke, Khoza-Shangase, & Kanji, 2019; Tomblin et al., 2015). It is therefore important that EHDI receives increased research focus to aid appropriate planning and budgeting for the South African health department. This planning should include efficient and effective screening protocols to ensure that NHS is conducted in a valid, reliable and ethical manner. Hence, the current study aimed to describe the outcomes of NHS at an academic secondary level hospital in Johannesburg, South Africa. The rationale of the study is to contribute towards contextually relevant evidence that facilitates efficacious provision of EHDI services in the South African context, particularly from a mother and child hospital setting where it is assumed early detection and intervention would be higher on the priority list than in a general hospital. Early detection of hearing loss is the initial stage to any EHDI programme and is conducted by means of NHS, the focus of the current study.

## Methods

### Study design

This study was part of another study programme titled ‘Universal Newborn Hearing Screening in Public Healthcare in South Africa: Challenges to Implementation’ (Bezuidenhout et al., 2018), where the design was a prospective non-experimental cohort study that looked at the feasibility assessment of a UNHS programme. In that study, the focus was on identifying challenges to implementation of the screening programme. The current study examines parameters that were being assessed. These included the time taken to screen, the risk factor profile of the newborns, the otoscopic examination results, as well as the follow-up rates during the programme. For this part of the study, the parameters being investigated were specific to the outcomes of the screening. Some of the findings, presented and discussed in depth in this study, were published in the Bezuidenhout et al.’s (2018) study.

### Study population and sample

The study population was drawn from all neonates born at Rahima Moosa Mother and Child Hospital (RMMCH), an academic secondary level hospital in Johannesburg, South Africa, using stratified systematic sampling. Selected neonates from the postnatal wards, the neonatal unit and neonatal intensive care unit (NICU) were assessed during a 3-month period.

Because of the limitation of availability of only one audiologist to screen the babies, a task they took on in addition to their clinical caseload, a stratified sample of neonates was recruited. At the time of the study, the hospital had a delivery rate of 20–30 babies a day, with a Caesarean section rate of 30%. By selecting 30% of the neonates to be tested from the Caesar theatre birth register and the remaining 70% from the labour ward register, a representative sample of the delivery profile specific to the testing hospital was ensured. A total of 10 neonates were identified every day to be screened, by selecting every third neonate appearing on the registries. This stratified, systematic sampling was done at the start of each weekday by the researcher, who assigned a study number to each of the pre-identified neonates requiring screening.

### Inclusion criteria

- Any neonate born at RMMCH within the specific 3-month period.
- At the time of screening, infants were to be younger than 30 days of chronological age to minimise the influence of extraneous variables such as hearing loss because of other causes, as well as residential location changes.

### Exclusion criteria

- Neonates who spent more than a month in NICU were excluded as they would have exceeded the age cut-off of 1 month.
- Any neonate whose parent/caregiver refused to provide informed consent.
- Neonates not born at the hospital site (transferred from other facilities).

### Study procedure

Hearing screening took place at the secondary academic hospital during weekday working hours by the audiology department team, comprising four audiologists who are registered with the HPCSA as being qualified to conduct all measures included in this study a part of their regulated scope of practice. One audiologist was assigned to screen each day, and this audiologist would receive a list of 10 names from the researcher and would attempt to screen as many neonates as possible from the list. The audiologist would explain the purpose of the screening to the caregiver, both verbally and via an information sheet. If the caregiver was in agreement, written informed consent was obtained prior to the hearing screening. Once informed consent had been obtained, the audiologist would note the starting time so that the duration of the screening process could be recorded.

Each neonate who was screened first underwent an otoscopic examination to assess patency of the ear canal as this could potentially impede the screening procedure and impact the results. Thereafter, distortion product otoacoustic emission (DPOAE) screening was conducted through the use of a Natus Bio-logic AuDX® device, giving either a ‘pass’ or ‘refer’ result. For contextual relevance, although inclusion of automated auditory brainstem response (AABR) audiometry would have been ideal and is an important part of a two-stage screening protocol, AABR is not readily available in most South African healthcare contexts, but OAEs are becoming increasingly so (Kanji & Khoza-Shangase, 2018a; Kanji, Khoza-Shangase, & Moroe, 2018), and AABR was not available at the research site. The same motivation is proffered for the use of DPOAEs instead of TEAOEs. Kanji and Khoza-Shangase (2018a) discuss differences in screening measures in various contexts and populations with TEAOEs being the most commonly used of the two measures. However, DPOAEs are also widely used in places such as the United States of America, where DPOAEs are second to AABR in frequency of use. China has also been reported to use DPOAEs within the different stages of the hearing screening protocol; therefore, the sensitivity of this measure in this population is not questionable (WHO, 2010). A ‘pass’ result was recorded if the patient passed the DPOAE test across at least 60% of the tested frequencies (1000 kilohertz [kHz], 2000 kHz, 3000 kHz, 4000 kHz and 5000 kHz) at 25 decibels (dB) – 30 dB hearing level, in both ears, where response level relative to the noise floor signal-to-noise ratio (SNR) should be at least 6 dB, with a minimum response level of −5 dB to −8 dB SPL and an acceptably low noise floor (−4 dB sound pressure level [SPL] or less) (Barker, Lesperance, & Kileny, 2000; Iowa Hearing Detection and Intervention Program, n.d). The current study included 1000 hertz (Hz) in the analysis in an attempt to cover lower frequencies. A ‘refer’ result implies that the patient did not pass the hearing screening test across at least 60% of the tested frequencies (1000 kHz, 2000 kHz, 3000 kHz, 4000 kHz and 5000 kHz) at 25 dB – 30 dB hearing level, in both ears.

Babies receiving a ‘pass’ result were discharged without a planned follow-up, unless follow-up was clinically indicated. It is acknowledged that discharging a baby after a *pass* on the initial screening may have resulted in false-negative findings which cannot be accounted for in this study; however, the resource allocations did not allow for the alternative. Newborns receiving a ‘refer’ result were rescreened within a month of initial screening. The rescreening procedures included an otoscopic examination, a tympanogram and a repeat DPOAE. If the results of the rescreening procedures were still inconclusive, the infant was referred for diagnostic auditory brainstem response (ABR) performed at a referral tertiary academic hospital.

Healthy neonates were screened within the first few days of life, whilst neonates with complications preventing earlier screening were screened when possible within the first 30 days of life. The neonates’ clinical history was obtained in all cases prior to the screening. This was obtained both verbally from the parent through an informal interview once consent had been given and by obtaining information documented in the neonate’s hospital file. A study data sheet was completed for each neonate that was screened. The study data sheet comprised three sections which included general information of the baby, the presence of risk factors and the findings on clinical assessment.

Firstly, *the general information section* provided information regarding the demographics of the babies being screened and included type of delivery, birth weight, HIV exposure, Apgar scores, as well as ward where the neonate was located. Secondly, *a risk factor assessment* was completed for each neonate screened. The following items were included: a family history of permanent childhood hearing loss; admission to NICU, and if so, was assisted ventilation required; exposure to ototoxic drugs such as aminoglycosides or loop diuretics; hyperbilirubinemia requiring exchange transfusion; congenital infections such as cytomegalovirus, herpes, toxoplasmosis, rubella, syphilis, HIV and malaria; and the presence of craniofacial abnormalities (HPCSA, 2018). Lastly, *examination of findings on clinical assessment section* where all data from the initial screening as well as the rescreening procedure were recorded.

### Data analysis

Data were entered into Microsoft Office Excel©, and later analysed using STATA intercooled version 11© (StataCorp, 2009). Data were analysed using both descriptive and inferential statistics. A Fisher’s exact test was used to assess the association between the risk factors and DPOAE results, with significance established where *p*-values less than 0.05 were considered statistically significant (Bonita, Beaglehole, Kjellström, & World Health Organization, 2006).

### Ethical consideration

The study adhered to the Singapore Statement on Research Integrity guidelines in terms of research into human subjects (Lucas, 2010). Therefore, prior to the commencement of the study, ethical approval was obtained from the University of Witwatersrand’s Human Research Ethics Committee (Protocol number M111119).

## Results and discussion

### Study population

During the 3-month study period, 2740 neonates were born at RMMCH, with a total of 490 neonates being identified and assigned study numbers. Of the identified neonates, two mothers refused consent for their infants to be screened. Because of the challenges discussed in Bezuidenhout et al. (2018), only a total of 121 neonates were screened, representing 24% of the identified neonates. Thus, of the 2740 neonates born at RMMCH during the 3-month study period, only 4.4% underwent NHS (Bezuidenhout et al., 2018).

**FIGURE 1:**
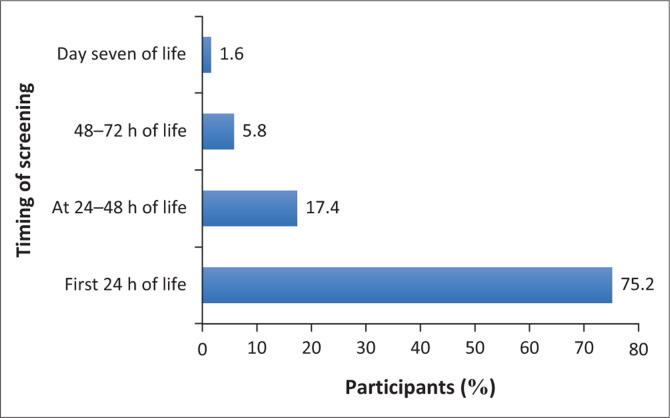
Time when hearing screening occurred in the current sample (*N* = 121).

Figure 1 depicts the time when hearing screening was conducted for each neonate included in the study. A large majority, 91 (75%), of the 121 neonates were screened in the first 24 h of life. Only two (1.6%) were screened on day 7 of life. The fact that many of the neonates were screened within the first 24 h is a positive finding as it indicates that hearing screening can be performed prior to discharge from the hospital. This is particularly important in the South African context where mothers and their neonates may be discharged home as early as 6 h post-normal vaginal delivery (NVD), according to the South African Department of Health Guidelines for Maternity Care (DoH, 2015). This, however, is only a positive finding if confounding variables to screening, such as the presence of vernix caseosa and the use of a two-stage approach, are actively addressed prior to the screening being conducted. The mean time taken to screen each neonate was 11 min 17 s, with the longest duration being 40 min, and the shortest screening time being 5 min. The median time taken to screen was 10 min. Technical difficulties with the DPOAE machine, the presence of vernix caseosa and high ambient noise were the main contributors to the prolonged screening time (Bezuidenhout et al., 2018).

### Hearing screening results

#### Otoscopic examination results

**FIGURE 2:**
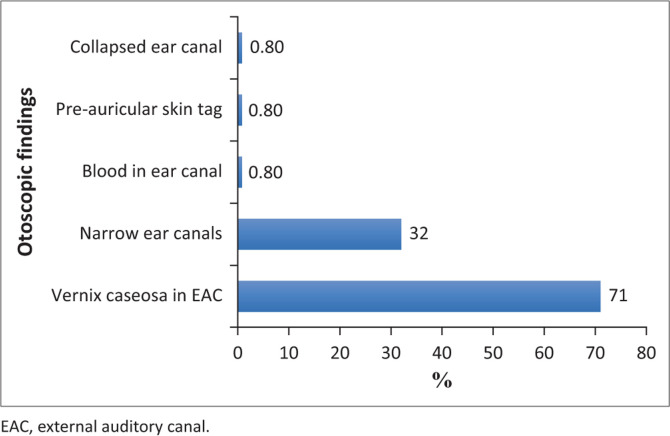
Otoscopic findings in neonates prior to hearing screening (*N* = 121).

Otoscopic examinations revealed a large majority, 86 (71%) neonates, had vernix caseosa in their external auditory canals, and 39 (32%) were subjectively considered to have narrow ear canals (Figure 2). Other otoscopic findings included the presence of blood in the ear canal (*n* = 1), a pre-auricular skin tag (*n* = 1) and an ear canal that collapsed during testing (*n* = 1).

The occurrence of a narrow ear canal was a subjective finding identified on otoscopic examination in 39 cases; 18 (46.2%) of which required a repeat DPOAE testing versus 39 (47.6%) of those without a narrow canal (odds ratio [OR]: 0.86, 95% confidence interval [CI]: 0.46–1.61, *p* = 0.88) – on additional statistical analysis.

Ear canal clearance was not performed on the participants because the study was meant to reflect the reality of the screening context. However, without artificial intervention, the high prevalence of vernix caseosa in the external auditory canal and its well-documented impact on OAEs raises important implications for the practicing audiologists and/or anyone involved in hearing screening, particularly in the first 24 h of life (Doyle, Rodgers, Fujikawa, & Newman, 2000; Kumari & Rangasayee, 2016). For efficient implementation of UNHS, neonatal assessment and management protocols that include standard manual otoscopy as well as compulsory external ear canal cleaning (vernix caseosa clearing) prior to OAE screening need to be considered. This is particularly important in the South African context where discharge can be as early as 6 h post-birth (DoH, 2015). This inclusion in screening protocols will improve efficiency of UNHS significantly, possibly reduce the high *refer* (false positives) rates and consequently reduce costs related to repeat screening which include emotional costs to parents when *refer* findings have been communicated to them.

#### Distortion product otoacoustic emissions

Of the total sample, 57 of the 121 participants (47%) had a *refer* result at the initial screening, whilst the remainder were discharged from the programme because they *passed* the screening bilaterally. All neonates that had *refer* findings were booked for a repeat DPOAE screening with only 20 (35%) of them returning for follow-up. Of the 20 that returned for repeat screening, all presented with normal otoscopy and tympanometry results, with only two presenting with *refer* findings on DPOAE screening. These two were referred for diagnostic testing including ABR testing. The tympanometry findings were all clearly defined single peaks type A tympanograms (Carmo, Costa, & Momensohn-Santos, 2013). Tympanometry data were not captured at the initial screening because of the unavailability of high frequency probe tone (1000 Hz) tympanometry at the time, which is the measure that has high sensitivity and specificity in this population (Carmo et al., 2013). This is a significant well-documented challenge with South African audiology practices which requires serious attention (Sebothoma & Khoza-Shangase, in press).

The two infants that were to be referred for diagnostic ABR measurements could not be tested because of technical issues with the ABR equipment. This resulted in a third DPOAE being conducted on the one infant who returned for the follow-up. This infant passed DPOAEs bilaterally, whilst the second infant defaulted follow-up. The rate of true versus false positive results could not be determined in the current sample. This is another important finding for this context where loss to follow-up can negatively influence the outcomes of an already compromised hearing screening programme.

Repeat testing was required for 57 (47%) neonates; however, only 20 infants returned for repeat DPOAE. This referral rate of 47% after initial screening is much higher than the HPCSA’s recommendation of <5% (HPCSA, 2018), which is in agreement with the guidelines from the JCIH that less than 4% of all newborns should fail the initial screening and be referred for repeat screening (JCIH, 2007). The target referral rate is a means of applying quality control to the screening programme. The high refer rates may have been attributed to inappropriate testing circumstances with excessive ambient noise interference, the presence of vernix and the faulty DPOAE machine. At least two of these contributing factors can be remediated to improve the referral rate. For example, a dedicated quiet room can be allocated for hearing screening in the wards with constant noise-level monitoring during screening. In addition, protocols can be put in place where vernix caseosa is routinely removed from the neonates’ ears as soon as it is safe to do so to facilitate screening via OAEs. The World Health Organization recommends 6 h as the earliest safe time when vernix can be removed (WHO, 2018). The high referral rate of 47% also contributed to increasing the burden of work for the under-resourced screening team. The return rate of those who require further diagnostic evaluation after failing the initial screening should be at least 95% (Kanji & Khoza-Shangase, 2018b). This highlights the importance of increasing all efforts to ensure that when screening happens, a two-stage approach is adopted in order to minimise the high referral rate.

#### Risk factor assessment

Of the total sample of neonates screened, two (1.7%) had been admitted to NICU and both had required assisted ventilation. The first neonate had an NICU stay of 6 days and passed the initial DPOAE which was conducted on day 7 of life. The second neonate was admitted to NICU for 8 days and was considered high risk for a possible hearing deficit as several risk factors were identified, namely:

- a positive family history (the maternal uncle) of permanent childhood hearing impairment
- the presence of a congenital syphilis infection
- intermittent positive pressure ventilation in NICU for 6 days
- postnatal exposure to aminoglycoside antibiotics.

In this neonate, the initial DPOAE result was that of a bilateral *refer*, but upon screening after discharge from NICU the results were a bilateral pass.

Other risk factors identified in the screened population included a family history of a permanent childhood hearing loss recorded in 10 (8.3%) neonates. Four of the neonates with a positive family history had a *refer* result on their initial DPOAE, and the caregivers were requested to return for follow-up testing. Only one infant was brought back for a second DPOAE, which was passed successfully. The remaining three infants were lost to follow-up.

Exposure to ototoxic medications in the form of aminoglycosides was recorded in three (2.5%) neonates in the screened cohort. One of these neonates with ototoxic drug exposure required a repeat DPOAE, which was successfully passed.

One neonate born at full term with a birthweight of 2.8 kilograms (kg) had a raised bilirubin level of 332 micromole per litre (µmol/L) at 72 h of life. According to the NICE guidance for neonatal jaundice, phototherapy was all that was required as the management (NICE Guidance, 2010). Although the initial DPOAE generated a *refer* result, the infant passed the repeat DPOAE.

Table 1 displays the risk factors according to the two groups – those that passed the initial DPOAE (*n* = 64) and those requiring a repeat test (*n* = 57).

**TABLE 1:** Outcome groups on initial distortion product otoacoustic emissions screening and recorded risk factors.

Variable	‘Passed’ screening test (*N* = 64)		Requiring repeat DPOAE (*N* = 57)		Total screened (*N* = 121)		*p*
	*n*	%	*n*	%	*N*	%	
Admitted to NICU	1	1.5	1	1.7	2	1.6	0.99
Family history of permanent childhood hearing loss	6	9.3	4	7	10	8.2	0.75
Exposure to ototoxic drugs	2	3.1	1	1.7	3	2.4	0.99
Hyperbilirubinemia	0	0	1	1.7	1	0.8	0.47
Congenital infection	0	0	1	1.7	1	0.8	0.47
HIV exposure positive	15	23	14	24.5	29	24	0.99

HIV, human immunodeficiency virus; NICU, neonatal intensive care unit; DPOE, distortion product otoacoustic emissions.

Findings depicted in Table 1 indicate no significant relationship between *refer* findings on DPOAEs and risk factors. However, the small sample size was a limitation to the interpretation of the risk factors as it relates to history, risk factors and actual conditions.

Although current screening findings indicate no significant relationship between the *refer* findings and risk factors, it is important to note that the screening protocol only included peripheral hearing screening tools in the form of OAEs and could have missed retrocochlear hearing impairment such as auditory neuropathy which can only be identified by ABR. Despite this limitation, current findings raise a need for interrogation of the relationship between risk factors and hearing impairment, as well as investigations on risk factors per specific context. Targeted screening programmes where risk factors were developed by organisations based in resource-rich nations may not identify risk factors which are prevalent in resource-scarce countries, for example, infectious diseases, non-elective caesarean delivery, maternal hypertension and malnutrition (Olusanya, 2011). A recent South African study by Le Roux, Swanepoel, Louw, Vinck and Tshifularo (2015) retrospectively reviewed 264 paediatric patients who had received cochlear implants and assessed the diagnosis and associated risk factors. They reported that a positive family history of a permanent childhood hearing loss, admission to NICU and prematurity were significant risk factors for profound hearing loss. Although these risk factors were present in the current study’s screened cohort, no neonates were found to have impaired hearing. The infant who defaulted on the third OAE had no significant risk factors on history, and was a well full-term neonate who had been delivered vaginally.

## Conclusion

Findings from this study revealed challenges with conducting a NHS programme in a South African academic secondary level mother and child hospital. Current findings were influenced by three key factors: (1) the capacity versus demands in as far as insufficient number of audiologists available to provide hearing screening at the facility, (2) the high rate of false positive test results which were influenced by vernix as well as the fact that only one-stage screening protocol was used and (3) the unacceptably high rates of loss to follow-up (Bezuidenhout et al., 2018). Of the 121 neonates screened, the majority (75%) were screened in the first 24 h of life. A large majority (71%) of these neonates presented with vernix caseosa on otoscopic examination. A high *refer* rate (47%) of the total sample was found on DPOAE screening. No maternal or neonatal risk factors were found to be significantly associated with *refer* findings in the screening programme. All these findings are not new for the South African context; however, they are when one considers that this is a hospital dedicated to mothers and children – where resource allocation and service delivery models adopted should be geared to be conducive to early detection and intervention.

The findings of this study raise implications for the implementation of NHS programmes in the South African context. Firstly, the staffing challenge needs to be addressed by possibly increasing the working hours of audiologists in the public healthcare sector to include evenings and weekends as babies are born and discharged during these times too. This is particularly important in a mother and child hospital facility. This, on its own, will not address the capacity versus demand challenge. However, when used in conjunction with task shifting this may significantly increase screening coverage. Because of the limited number of audiologists in the country, non-audiologists (including volunteers and/or nurses) should be trained to be screeners with supervision provided by audiologists in this task-shifting model of care. Secondly, as part of the screening programme, removal of vernix caseosa from the external auditory meatus needs to be done routinely in order to ensure that this does not become a confounding variable in the screening findings. This is particularly important as often otoscopic examination does not routinely form part of standard screening protocols. Because of the early discharge and the high likelihood of the presence of vernix caseosa within the South African context, inclusion of otoscopic examination in the screening protocol has been demonstrated to be important. Thirdly, repeat screening for all neonates with *refer* findings following vernix caseosa removal should be done before discharge to ensure reduction of the high *refer* (false positives) rates that have negative impact of parental anxiety as well as on resource use. Fourthly, establishment of reasons for poor return rates for follow up need to be investigated for this context, and solutions put in place as return rate is important for success of any screening and intervention programme. One key recommendation, to ensure that follow-up appointments for repeat screening and/or diagnostic testing are aligned with other medical follow-up services, should be consistently adhered to in screening programmes (Kanji & Khoza-Shangase, 2018b). Fifthly, the South African audiology community should lobby for a national political mandate of UNHS by the South African government to facilitate strategic implementation and monitoring of hearing screening programmes as part of mandated early childhood intervention programmes such as The First 1000 days campaign. Lastly, planning of screening programmes in the South African context should consider continuity of care, which comprises availability of functional diagnostic audiological equipment for confirmation of screening findings.

Current findings must be interpreted taking into consideration the identified methodological limitations. The 3-month time period used for data collection was the main limitation of the study. It is believed that a longer time frame where other variables could have come into play might have influenced the findings of the study (Bezuidenhout et al., 2018). Secondly, the study was in an academic hospital in Johannesburg where resources are significantly better than in several other hospitals in the country, therefore limiting the generalisability of the findings. Lastly, the small sample size has an influence on the generalisation of the findings and raises implications for future studies. It is important therefore that current findings be interpreted with these limitations in mind, and that future studies consider these in their study designs. Nonetheless, these findings add to the contextually relevant evidence from the South African context, and raise implications for clinical planning as well as strategic planning around when screening should be conducted, what measures to put in place to improve efficiency of hearing screening programmes, the importance of investigating and mitigating poor return rate to follow-up, as well as investigations of the validity of risk factors to hearing impairment – and the implications of these in hearing screening programmes.
